# Tau and neuroinflammation in Alzheimer’s disease: interplay mechanisms and clinical translation

**DOI:** 10.1186/s12974-023-02853-3

**Published:** 2023-07-14

**Authors:** Yijun Chen, Yang Yu

**Affiliations:** Shanghai Frontiers Science Center of Drug Target Identification and Delivery, Engineering Research Center of Cell and Therapeutic Antibody, Ministry of Education, School of Pharmacy, Shanghai Jiao Tong University, Shanghai, 200240 China

**Keywords:** Alzheimer’s disease, Tauopathies, Tau phosphorylation, Tau propagation, Neuroinflammation, Glia, Microglia, Astrocytes

## Abstract

Alzheimer’s Disease (AD) contributes to most cases of dementia. Its prominent neuropathological features are the extracellular neuritic plaques and intercellular neurofibrillary tangles composed of aggregated β-amyloid (Aβ) and hyperphosphorylated tau protein, respectively. In the past few decades, disease-modifying therapy targeting Aβ has been the focus of AD drug development. Even though it is encouraging that two of these drugs have recently received accelerated US Food and Drug Administration approval for AD treatment, their efficacy or long-term safety is controversial. Tau has received increasing attention as a potential therapeutic target, since evidence indicates that tau pathology is more associated with cognitive dysfunction. Moreover, inflammation, especially neuroinflammation, accompanies AD pathological processes and is also linked to cognitive deficits. Accumulating evidence indicates that inflammation has a complex and tight interplay with tau pathology. Here, we review recent evidence on the interaction between tau pathology, focusing on tau post-translational modification and dissemination, and neuroinflammatory responses, including glial cell activation and inflammatory signaling pathways. Then, we summarize the latest clinical trials targeting tau and neuroinflammation. Sustained and increased inflammatory responses in glial cells and neurons are pivotal cellular drivers and regulators of the exacerbation of tau pathology, which further contributes to its worsening by aggravating inflammatory responses. Unraveling the precise mechanisms underlying the relationship between tau pathology and neuroinflammation will provide new insights into the discovery and clinical translation of therapeutic targets for AD and other tau-related diseases (tauopathies). Targeting multiple pathologies and precision therapy strategies will be the crucial direction for developing drugs for AD and other tauopathies.

## Background

Dementia is currently one of the leading causes of disability and dependency among the elderly [1]. The World Health Organization reported that in 2022, more than 55 million people live with dementia worldwide, which will increase to 139 million by 2050 [2]. Dementia has become a major public health challenge and imposes enormous societal and economic burdens. Alzheimer’s disease (AD) contributes to 60–70% of dementia cases and is characterized by poor learning and memory as well as progressive and irreversible declines in cognition and behavior [3]. The two prominent pathological hallmarks of AD are the extracellular neuritic plaques (NPs) and intercellular neurofibrillary tangles (NFTs) consisting of the accumulation of β-amyloid (Aβ) and hyperphosphorylated tau protein, respectively [4]. In addition, activation of inflammatory processes and immune responses are commonly observed in AD brain tissues [5].

As an age-related neurodegenerative disease, AD is divided into two subtypes according to the age of onset: early-onset AD (EOAD, < 65 years) and late-onset AD (LOAD, ≥ 65 years). Most AD cases are LOAD, a complex disease with heterogeneous etiologies including genetics, aging, environment, lifestyle, and chronic diseases, such as obesity [6]. EOAD accounts for about 10% of the total AD cases. Only 5% of patients with EOAD carry a pathogenic variant in the AD genes (*APP*, *PSEN1*, and *PSEN2* coding for the amyloid precursor protein, the presenilin 1 and 2, respectively) or the apolipoprotein E (*APOE*) ε4 allele. The pathogenesis remains unknown in most patients with EOAD [7].

Although the symptoms of AD are well-studied, no treatments can halt and reverse the progression of AD. Our understanding of AD pathogenesis is still limited. At present, the pathogenic hypotheses of AD mainly include the amyloid cascade hypothesis, tau hypothesis, inflammatory hypothesis, cholinergic hypothesis, etc. The amyloid cascade hypothesis is the most widely accepted one. However, nearly, all anti-Aβ drugs have failed to show satisfactory therapeutic efficacy in the past two decades. It is encouraging that in 2021, aducanumab, a humanized recombinant monoclonal antibody targeting Aβ, became the first disease-modifying therapy (DMT) drug for AD approved by an accelerated pathway of the US Food and Drug Administration (FDA). This approval based on reduced amyloid markers and its clinical efficacy remains controversial [8, 9]. In January 2023, FDA approved a new monoclonal antibody against Aβ called lecanemab for the treatment of early AD, also by its accelerated approval pathway. Lecanemab reduced brain amyloid burden markedly in early AD and cognitive decline moderately than placebo at 18 months but was related to adverse events [10]. The efficacy or long-term safety of these two drugs needs further validation. In addition, it is timely to revisit the amyloid cascade hypothesis and consider other targets, such as anti-tau or anti-inflammatory drug development for AD. Increasing evidence demonstrates that the cognitive dysfunction and severity of the disease are more related to tau pathology [11–14]. The inflammatory responses (especially neuroinflammation) accompany the entire progress of AD pathogenesis and are also linked to cognitive dysfunction [15]. Therefore, this review focuses on tau pathological changes in the progression of AD and summarizes recent studies on the mutual regulation and influence of neuroinflammation and tau pathology. The current clinical drug development based on the tau and inflammation hypotheses is also discussed. This review will provide new insights into AD pathogenesis and drug treatment strategies.

## Mechanisms of tau-mediated neurodegeneration

Neuronal inclusions composed of the aberrant aggregated microtubule-associated protein tau (MAPT) have been found in the brains of patients with neurodegenerative disorders called tauopathies, including AD, progressive supranuclear palsy (PSP), frontotemporal lobar degeneration (FTLD), and Pick’s disease [PiD, also termed frontotemporal dementia and parkinsonism linked to chromosome 17 (FTDP-17)]. Misfolded tau is a key pathological feature in AD, the most common tauopathy. The insoluble tau deposits comprised of fibrils are most commonly found in the cell bodies and dendrites of neurons, and they are called NFTs [16]. Correlations between NFT density and clinical symptoms, such as a cognitive decline in AD, have been demonstrated [11, 12].

### Expression and function of tau

Tau was first discovered in 1975. As a microtubule-associated protein, it is expressed at a high and soluble level in neurons throughout the central nervous system (CNS) [17]. Tau is predominantly found in the axons of neurons. A pivotal function of tau is to bind to microtubules, enhance the assembly, and regulate the stability of microtubules, which plays essential roles in neurite outgrowth, cell shape and polarity, and intracellular cargo (such as neurotransmitters) transport [18].

The human tau is encoded by the 16 exons-comprising MAPT gene on chromosome 17q21 [19]. In the human brain, alternative splicing of exons 2 and 3 of the tau gene produces three isoforms with 0, 1, or 2 N-terminal repeats (0N, 1N, 2N), whereas the absence or presence of exon 10 results in tau species with either three (3R) or four (4R) carboxyl-terminal microtubule-binding domain. Thus, six major tau isoforms are expressed in the human brain and range from 352 to 441 amino acids in length [19]. The expression of Tau isoforms is regulated developmentally. In the normal adult brain, all six isoforms are present with approximately equimolar 4R and 3R isoforms, whereas, in the human fetal brain, only 0N3R tau is expressed [18]. The 4R tau isoforms exhibit higher affinity when binding to microtubules than the 3R isoforms [20]. Studies have shown that some known mutations in the tau gene affect the alternative splicing of exon 10, resulting in an altered 4R:3R ratio, a crucial feature of primary tauopathies [21, 22]. Primary tauopathies are a subgroup of FTLD disorders characterized by neuronal and glial tau inclusions with predominant frontal and temporal lobe atrophy. According to the fibrillated tau isoform (3R or 4R), primary tauopathies can be further classified into three major subtypes, including 3R tauopathies (such as PiD), 4R tauopathies (including PSP and corticobasal degeneration (CBD)), and mixed 3R/4R tauopathies [23]. AD is considered a secondary tauopathy due to tau pathology may occur as a consequence of extracellular amyloid plaques. In AD brains, tau aggregates into NFTs or neuropil threads composed of 3R and 4R Tau [24]. In this review, we focused on AD, the representative of secondary tauopathies.

Insoluble fibril formation of tau has long been considered an essential toxic event in AD. However, numerous studies have shown that the smaller, soluble, and non-fibrillar tau oligomers, called the “tau we cannot see” [25], play a more critical role in the neurotoxicity and propagation of tau damage in the CNS [25–29]. Mutations in the MAPT gene lead to FTDP-17 [22], providing evidence that tau dysfunction due to tau mutations induces neurodegeneration. Recent studies have found that overexpression of either wild-type tau or human P301L-mutant tau inhibits neural network activity independent of fibril formation. Turning off their overexpression attenuates the inhibition of network activity, which is associated with soluble tau but not fibrillar tau [30]. Furthermore, inhibition of endogenous tau improves behaviors and protects neurons from toxicity in APP/PS1 mice, a mouse model with AD-like Aβ pathology [31]. These findings indicate that soluble oligomeric tau may play a more essential role in neurodegeneration than insoluble fibrillar tau (including NFT), the “tau we can see”.

### Post-transcriptional modifications of tau

In humans, tau protein undergoes several post-translational modifications to regulate the interactions with microtubules, including phosphorylation, N-linked glycosylation (N-glycosylation), O-linked N-acetylglucosaminylation (O-GlcNAcylation), glycation, ubiquitination, truncation, nitration, and oxidation [19]. Phosphorylation is the most widely studied post-translational modification for tau, as more than eighty serine and threonine residues and five tyrosine residues are potential phosphorylation sites on the longest isoform of human tau [32]. The normal phosphorylation state of tau is critical for neuronal plasticity.

However, under pathological conditions, various highly increased post-translational modifications, such as hyperphosphorylation, destabilize the interaction of tau with microtubules [33] and enhance the capacity of tau to accumulate in the cytoplasm [34], leading to microtubule instability and transport dysfunction. In the normal adult brain, there are 2–3 mol of phosphate per mole of tau, but in the AD brain, tau protein is twofold to threefold hyperphosphorylated [35]. Studies have shown that individual missense mutations in tau alter potential phosphorylation sites and promote phosphorylation levels compared to unmutated tau [36]. In addition, various kinases and phosphatases have been found to regulate tau phosphorylation, such as glycogen synthase kinase-3β (GSK3β), cyclin-dependent kinase-5 (CDK5), p38 mitogen-associated protein kinase alpha (p38α MAPK), extracellular signal-related kinase (ERK), c-Jun N-terminal kinase (JNK), protein kinase A (PKA), and protein phosphatase 2 (PP2A) [4, 37–39]. A recent study demonstrated that tau phosphorylation is controlled by interdependence, an initial site-specific phosphorylation (they called “master sites”) leads to subsequent multi-site phosphorylation. Co-targeting p38α, the most central tau kinase associated with interdependence, and the master sites synergistically eliminated hyperphosphorylation of tau [39]. Hyperphosphorylated tau leads to abnormal aggregation of tau protein, which loses its ability to stabilize microtubules, thereby impairing neuronal function [23]. In addition, tau aggregates have been shown to have prion-like seeding and spreading properties [40]. The pathogenic tau can be released from diseased neurons, then uptake by previously unaffected normal neurons, inducing pathogenic tau production in normal neurons. This property of pathogenic tau leads to disease progression and broader clinical symptoms [41, 42]. Further discussion of tau propagation is provided in the following section.

In addition to abnormal hyperphosphorylation, other types of post-translational modifications of tau may also contribute to tau dysfunction in disease states. For example, reduced tau *O*-glycosylation could lead to increased phosphorylation, while the enhancement of *O*-glycosylation reduces the extent of tau phosphorylation [43–45]. In addition, the acetylation of tau is an early pathological feature of neurodegeneration. Acetylated tau inhibits its degradation, promotes pathological aggregation and propagation, and contributes to tauopathy [46–51]. Other post-translational modifications, such as isomerization and truncation, have been shown to promote and stabilize paired helical filaments (PHFs) [52, 53]. The methylation of tau could suppress the aggregation of tau [54]. The precise mechanism of these post-translational modifications of tau in Alzheimer's neurofibrillary degeneration is unclear. However, the hyperphosphorylation alone can induce pathological functional changes in tau, promoting self-aggregation into PHF tangles. Moreover, tau hyperphosphorylation is present in almost every tauopathy, suggesting that different post-translational modifications may be involved in modulating hyperphosphorylation [4]. Therefore, targeting hyperphosphorylation of tau, which may be a convergent pathway of tauopathies, including AD, is a crucial direction for drug development.

### Propagation of tau pathology

Studies propose that AD and other non-infectious neurodegenerative disorders associated with aggregation of fibrillar proteins exhibit features similar to prion disease [55]. The transfer of abnormal misfolded proteins, including tau and Aβ, has a common feature of the pathological propagation between cells [42, 56]. Postmortem studies have shown that the spread of tau pathology in AD follows a predictable pattern, allowing neuropathological diagnosis of different AD stages defined by Braak staging (I–VI). Initial neurofibrillary tangles and neuropil threads develop in the entorhinal cortex, then the hippocampus, and gradually affect additional brain regions as the disease progress, eventually affecting the neocortex [57]. An in vivo study has demonstrated that the intracerebral injection of synthetic tau fibrils into the hippocampus or frontal cortex of tau-P301L transgenic mice at 3 months increases tau hyperphosphorylation and accumulation around the injection site. In addition, the spread of tau pathology is time-dependent from the injection site to distant interconnected brain regions [58].

**Fig. 1:**
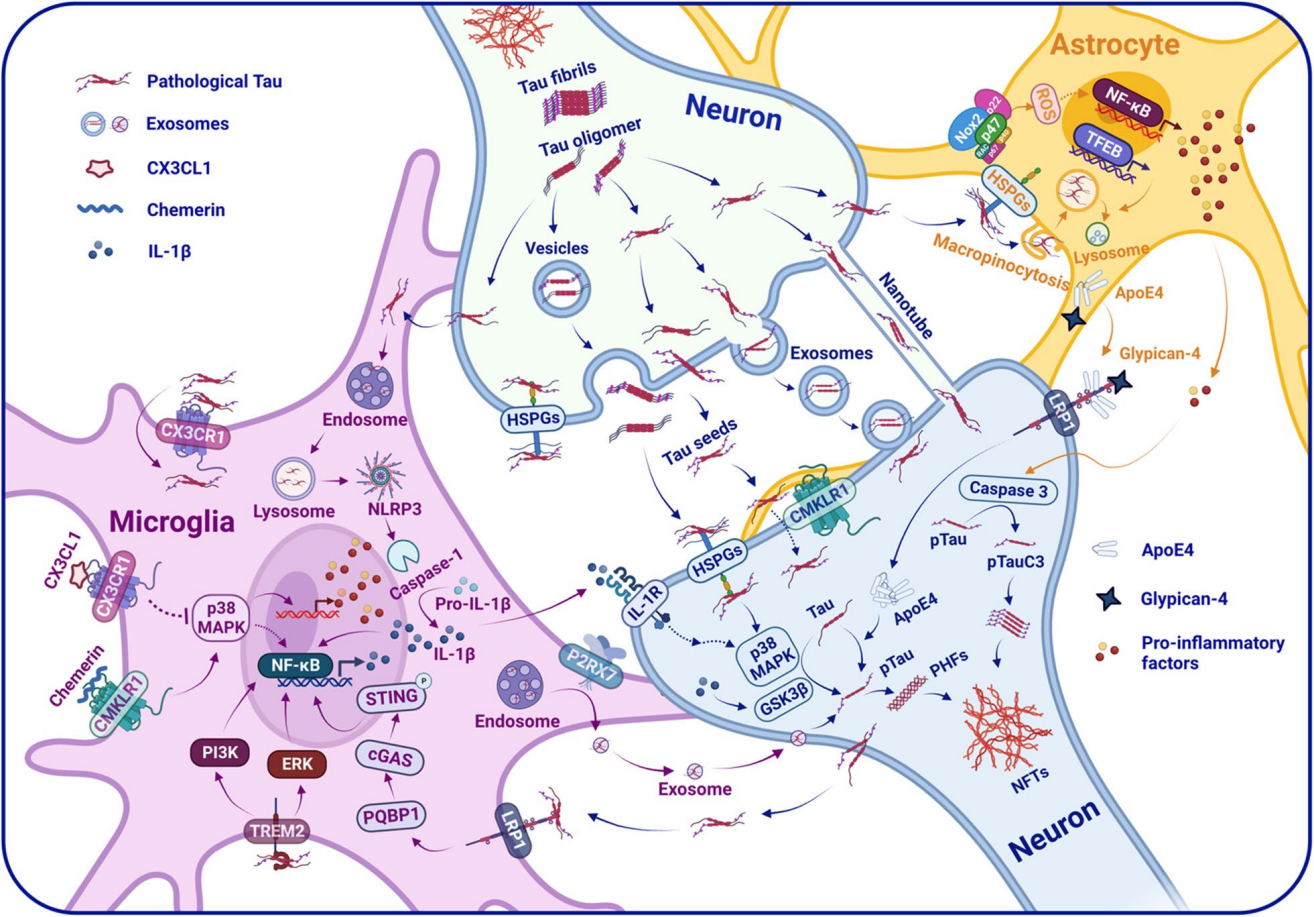
Schematic diagram showing the interaction between neuroinflammation and tau pathology contributing to the progress of AD pathogenesis. *Aβ* β-amyloid, *ApoE4* apolipoprotein E4, *cGAS* cyclic GMP–AMP synthase, *CMKLR1* chemerin chemokine-like receptor 1, *CX3CL1* chemokine (C–X3–C motif) ligand 1. *CX3CR1* CX3C motif chemokine receptor 1, *GSK3β* glycogen synthase kinase-3 beta, *IL-1β* interleukin-1β, *IL-1R* interleukin-1 receptor, *HSPGs* heparan sulfate proteoglycans, *LRP1* low-density lipoprotein receptor-related protein 1, *MAPK* mitogen-activated protein kinase, *NF-κB* nuclear factor kappa B, *NFTs* neurofibrillary tangles, *NLRP3* NLR family pyrin domain-containing protein 3, *Nox2* NADPH oxidase 2, *P2RX7* P2X purinoceptor 7, *PHFs* paired helical filaments, *PQBP1* polyglutamine-binding protein 1, *pTau* phosphorylated tau, *STAT1* signal transducer and activator of transcription 1, *STING* stimulator of interferon genes, *TFEB* transcription factor EB, *TNF-α* tumor necrosis factor α, *TREM2* triggering receptor expressed on myeloid cells 2

Although the specific mechanisms underlying the interneuronal spread of these tau aggregates remain poorly understood, there is a large amount of evidence indicating that the abnormally hyperphosphorylated or oligomeric tau can be secreted from neurons into the extracellular space, then be taken up by other normal neurons, and finally causes interneuronal transfer of tau pathology and spreading of tau toxicity across different brain regions [59–61] (Fig. 1). The protopathic tau seeds may be released and internalized by neurons through trans-synaptic and non-synaptic pathways in parallel to promote tau spreading [62–65]. Sokolow et al. revealed that C-terminal truncated tau is abundant in the cortical pre-synaptic terminals, and tau cleavage promotes tau aggregation, secretion, and propagation in AD [66]. In addition, trans-synaptic tau propagation and aggregation can also be independent of the presence of endogenous soluble tau, but the absence of endogenous tau reduces its neurotoxicity [63].

It has been proposed that intracellular tau can be released from neurons by exocytosis, including secretion (free protein), extracellular vesicles (such as exosomes and ectosomes/microvesicles), and neuronal death pathways [67]. Meanwhile, the extracellular tau can be taken up by neighboring cells, both neurons and glial cells, through various pathways, including phagocytosis [68], macropinocytosis [69], receptor-mediated uptake [67, 70], endocytosis [71], and/or membrane fusion of exosomes [72, 73]. A recent study showed that the low-density lipoprotein receptor-related protein 1 (LRP1) expressed in neurons regulates the endocytosis of tau and its subsequent spread [74]. Knockdown or inhibition of LRP1 significantly reduces tau uptake and spread in vitro and in vivo [74]. In addition, exosomes are involved in the dissemination of tau pathology. Exosomes isolated from the brain, cerebrospinal fluid (CSF), and plasma of AD patients and/or AD animal models contain pathologic Aβ and tau [73, 75–77]. Increased neuronal activity enhances the release of tau-containing exosomes [75] and exacerbates tau propagation and pathology [78]. Exosomes carrying tau are taken up by local and remote cells and contribute to apoptosis and neuronal loss [79]. Peeraer and colleagues found that the tau pathology as a consequence of injection with tau-preformed fibrils into the hippocampus of tau-P301L transgenic mice induced selective neuron loss of the CA1 region [58]. Inhibition of exosome synthesis using the neutral sphingomyelinase-2 inhibitor reduces tau propagation from the entorhinal cortex to the dentate gyrus in adeno-associated virus (AAV)-based and P301S tauopathy mouse models [80]. These findings reveal a pivotal role in the cell-to-cell spreading of abnormal tau in neurotoxicity and provide potential therapeutic strategies for tau-targeted immunotherapies in AD and other tauopathies. Furthermore, tau propagation-induced tau pathology is based on the spread of tau between neurons and the dissemination between neurons and glial cells [81, 82]. Glial cells such as microglia and astrocytes perform normal immune functions in CNS and also play a vital role in the spreading of pathological tau [80, 83]. This part will be discussed in detail in the next section.

## Neuroinflammation: a link between tau and AD

Over the last decade, evidence indicates that CNS inflammation (neuroinflammation) may play a pivotal role in the pathological progression of AD. The chronic, sustained inflammatory response in the brain is considered the third core pathological feature of AD. It provides a link between the other two core pathologies, Aβ plaques and NFTs [84]. The acute neuroinflammatory response is a fundamental protective immune response against noxious and irritable stimuli, such as infection, toxins, and injury, which is a well-established defense response and is essential for the brain's repair process. However, when the balance of pro-inflammatory and anti-inflammatory signaling is disrupted, it leads to a chronic inflammation response. This chronic neuroinflammation is caused by persistent activation of glial cells and excessive release of cytotoxic molecules, which adversely affects brain function and is a major cofactor in the pathogenesis of many neurodegenerative disorders, including AD [84].

The neuroinflammatory process in AD is mainly driven by the innate immune cells in the brain, including microglia and astrocytes [85]. Microglia are the resident immune cells of the brain and play a pivotal role in the immune defense of CNS. They are in an inactive “resting” state under physiological conditions while actively monitoring the brain environment and brain parenchyma with highly motile processes [86]. Microglia will shift from the resting status to an activated state when they recognize a stimulus in CNS, characterized by the morphological changes and modulations in the gene expression, including pro- and anti-inflammatory molecules and microglial surface receptors. These receptors include the triggering receptor expressed on myeloid cells 2 (TREM2), toll-like receptors (TLRs), and G protein-coupled receptors (GPCRs), such as chemokine CX3C motif receptor 1 (CX3CR1), formyl peptide receptor 2 (FPR2) and chemokine-like receptor 1 (CMKLR1), which upon activated by the stimuli, mediate microglial activation and polarization phenotype [87, 88]. Microglial activation is believed to be a double-edged sword in AD pathology. In the early stages of AD, activated microglia cause phagocytosis and clearance of pathologic Aβ and/or tau, positively affecting AD pathologies in animal models [89, 90]. However, sustained activation of microglia leads to the continuous release of inflammatory factors and reduces their ability to phagocytose and degrade neurotoxins, which in turn exacerbates Aβ accumulation, tau propagation, and neuronal death, ultimately promoting AD progression [5, 91, 92].

Recent evidence suggests that reactive astrocytes also contribute to neuroinflammatory processes associated with AD pathology [93]. Astrocytes are the most abundant glial cells in the brain and have a variety of complex and essential functions in CNS, including maintenance of brain homeostasis, synaptic transmission, and information processing through neural circuits [94]. Astrocytes become activated in AD and exhibit certain immune functions. Like microglia, astrocytes can be triggered by various factors, such as pathological Aβ, tau species, and proinflammatory cytokines [95, 96]. The activated microglia and reactive astrocytes produce nitric oxide (NO) and inflammatory cytokines, such as interleukin-1 (IL-1), IL-6, tumor necrosis factor α (TNF-α), and transforming growth factor β (TGF-β), that contribute to a reinforced inflammatory cascade [85, 97]. Studies have shown that these pro-inflammatory cytokines are markedly elevated in the brain and CSF of AD patients [98–100]. Interestingly, the upregulation of such proinflammatory cytokine has been observed even before signs of increased Aβ and hyperphosphorylated tau in CSF of mild cognitive impairment (MCI) patients [101], suggesting that the inflammatory processes had occurred in the early stages of AD.

Although the link between neuroinflammation and AD was discovered decades ago [102], it remains unclear whether it is a cause or a consequence of the disease. Recent studies have shown that microglial activation occurs in the preclinical AD stage. In addition, with the progression of the disease, immune activation, including the activation of microglia and astrocytes, diverts to a more harmful stage [103]. This indicates that neuroinflammation may be involved in the etiology of AD. Furthermore, since the immune response exists throughout the pathological progress of AD [104, 105], suggesting that it may also participate in and aggravate the disease development. In addition, not limited to neuroinflammatory response, systemic inflammation is currently beginning to be considered a contributor to AD development of AD [106, 107]. Multiple epidemiological studies show that anti-inflammatory drugs such as non-steroidal anti-inflammatory drugs (NSAIDs) have a sparing effect on AD [108–110]. Unexpectedly, clinical trials targeting inflammation with NSAIDs have not improved cognition in AD patients [111, 112]. The outcomes of clinical trials may be related to the disease stages of recruited AD patients. Moreover, these results also indicate that further elucidation of the exact relationship and mechanism between brain inflammatory events and the pathological development of AD is necessary to explore successful therapies and drugs.

Evidence suggests that the pathological activation of glial cells and the release of inflammatory factors involved in neuroinflammation can exacerbate tau pathology via direct or indirect pathways, leading to neuronal damage and cognitive impairment, ultimately aggravating AD pathology [113, 114]. The neuroinflammation exacerbating tau pathology may be correlated with the regulation of tau post-translational modification and propagation [115]. Modifying or intervening in the immune response to slow down or inhibit tau pathology provides a potential direction for developing potential therapeutic drugs for AD. In the succeeding sections, we discuss several possible mechanisms for the link between altered neuroinflammation and tau pathology observed in AD (Fig. 1). We highlight the potential value of targeting the combination of neuroinflammation and tau pathology and/or their link in AD treatment.

### Role of neuroinflammation in post-transcriptional modifications of tau

Accumulating evidence suggests that microglia activation participates in the progression of tau-related neuropathology. Felsky et al. reported that the proportion of morphologically activated microglia (PAM) in postmortem cortical tissue from AD patients is strongly related to tau pathology, and their mediation models support microglial activation as an upstream event in AD leading to accumulation of hyperphosphorylated tau and subsequent cognitive decline [116]. Our and other studies found that systemic administration of lipopolysaccharide (LPS, inducer of inflammation) leads to microglial activation in the mouse brain, which results in tau hyperphosphorylation at specific sites [117, 118]. The effect of microglial activation on tau hyperphosphorylation is related to cell surface receptors which mediate inflammatory responses.

TREM2, a pivotal risk factor for LOAD [119, 120], is associated with tau pathology. TREM2 is a receptor for Aβ [121] and is exclusively expressed by microglia in the brain of mice and humans [122, 123]. In the CSF of AD patients, the R47H (rs75932628) variant of TREM2 or soluble TREM2 has been found to correlate with total or phosphorylated tau (Thr181), respectively, but not with Aβ_42_ [124, 125]. Numerous studies have been conducted in animal models to explore how TREM2 affects tau pathology. TREM2 deficiency in a hTau (expressing human *MAPT* but not endogenous mouse *Mapt*) mouse model exacerbates tau phosphorylation and aggregation at the early disease stage [126]. In contrast, in tau-P301S transgenic mice, TREM2 deletion attenuates neuroinflammation and protects against neurodegeneration at a late stage without altering tau phosphorylation and aggregation [127]. Further research reveals that only in the presence of Aβ pathology, TREM2 deletion further exacerbates tau accumulation and brain atrophy [128]. TREM2 may play a pivotal role in all stages of AD pathogenesis, and maintaining the normal function of TREM2 may point out a direction for AD treatment.

CX3CR1, another receptor explicitly expressed on microglia, is involved in tau pathology. CX3CR1 belongs to G protein-coupled receptors (GPCRs). GPCRs, as one of the most prominent protein families, is a class of receptors with seven-transmembrane domains. GPCRs sense extracellular molecules and then transduce the signals to intracellular effector molecules, resulting in cellular responses [129]. A body of evidence indicates the opposing effects of CX3CR1 with its ligand fractalkine (CX3CL1) on Aβ and tau pathologies. Deletion of CX3CR1 or depression of the CX3CL1/CX3CR1 axis reduces Aβ deposition [130–132] but exacerbates tau pathology, such as increased phosphorylation and aggregation of tau, and this is associated with worsened behavioral and cognitive impairments [132–137] (Fig. 1). These findings suggest that regulating the CX3CL1/CX3CR1 axis may be a potential target for preventing tau-related neurodegeneration.

In addition, astrocytes are also suggested to play a role in the hyperphosphorylation of tau. Astrocytes exacerbate Aβ-induced tau hyperphosphorylation and truncation. The mechanism relates to the increased caspase-3 activity caused by soluble inflammatory factors released by active astrocytes [96]. Our recent study shows that p47^phox^, the organizer subunit of NOX2 (Nicotinamide adenine dinucleotide phosphate oxidase 2, NADPH oxidase 2), is associated with cognitive function and tau pathology in AD. The expression of p47^phox^ in neurons contributes to tau hyperphosphorylation directly, while p47^phox^ in astrocytes affects tau hyperphosphorylation by activating astrocytes indirectly [13]. ApoE4, the most potent risk factor for the pathogenesis of LOAD, modulates neuroinflammatory response and glial activation [138]. Astrocyte- or neuron-specific ApoE4 could regulate tau phosphorylation in glia-dependent or independent manner [139–141]. Saroja et al. demonstrated that astrocyte-secreted ApoE4 and glypican-4 (GPC-4) bind to LRP1 in neurons, leading to tau accumulation and propagation [141] (Fig. 1). Deletion of astrocytic ApoE4 markedly reduces phosphorylated tau [142]. ApoE4 affects neuroinflammation, tau pathology, and tau-mediated neurodegeneration independently of Aβ pathology [143]. These findings indicate that astrocytes are crucial in exacerbating tau hyperphosphorylation and ultimately promoting AD pathology. In addition, tau is also present in the astrocytes of individuals with AD [144, 145]. However, how tau pathology is induced and regulated in astrocytes in AD and other tauopathies remains unknown.

FPR2 and CMKLR1, two GPCRs expressed on astrocytes and/or microglia, are also shown to be related to AD pathology. They are known initially as orphan receptors and recognize various endogenous and exogenous chemotactic ligands to exert pro-inflammatory or anti-inflammatory functions [146, 147]. Aβ is one of their ligands [148, 149]. Recently, the structure of the FPR2-Gi protein complexed with Aβ has just been solved [150]. Deficiency of FPR2 or administration of its inhibitors/anti-inflammatory ligands could alleviate the pathological symptoms of AD, including reduced activation of glial cells (microglia and/or astrocytes) and tau hyperphosphorylation, and ultimately leading to improvement in cognitive function [151–154]. Our recent study identified that CMKLR1 deletion increases Aβ plaques in the AD mouse brain but reduces mortality and cognitive deficits of AD mice and attenuates tau hyperphosphorylation [14]. Further studies found that CMKLR1 and its ligand chemerin regulate the migration and recruitment of microglia to Aβ plaques in vivo and in vitro [155]. Pro-resolving ligands or inhibitors of CMKLR1 have been shown to attenuate inflammatory responses, including neuroinflammation [156–158]. Whether FPR2 and CMKLR1 regulate the abnormal phosphorylation of tau by mediating microglial activation needs further verification.

The effect of microglia and astrocytes on tau pathology is related to the production and release of pro-inflammatory cytokines after their activation. A recent study shows that NLR family pyrin domain-containing protein 3 (NLRP3) inflammasome activation induces tau hyperphosphorylation and aggregation through modulating tau kinases and phosphatases [159]. NLRP3 inflammasome has been proven to accumulate inside microglia upon activation, promoting cleavage and activity of caspase-1 and release of IL-1β [160]. IL-1β treatment or overexpression exacerbates tau phosphorylation through the activation of p38 mitogen-activated protein kinase (MAPK) and/or glycogen synthase kinase-3β (GSK-3β) in neuron–microglia co-cultures [161] and AD model mice [162, 163]. Bhaskar et al. reported that microglia activation induces tau hyperphosphorylation via the IL-1β/p38α MAPK pathway in vitro and in vivo [135, 137]. Selective suppression of p38α MAPK significantly reduces tau hyperphosphorylation and improves working memory in hTau mice [164]. Our previous studies indicate that the deficiency of serum amyloid A (SAA), an acute-phase protein with cytokine-like properties, enhances tau phosphorylation induced by systemic LPS administration [117]. Overexpression of SAA by intracerebral injection attenuates tau hyperphosphorylation, and the mechanism is related to SAA-induced secretion of IL-10 from microglia [117]. Another study demonstrates that IL-10 deletion activates microglia, increases IL-6 production, and leads to tau hyperphosphorylation in response to acute systemic inflammation [165]. Other inflammatory factors such as IL-3, IL-6, IL-18, tumor necrosis factor-α (TNFα), and macrophage migration inhibitory factor (MIF) are also found to be involved in tau phosphorylation and/or truncation [166–170]. All these findings suggest that glia-neuron signaling contributes to the pathogenesis of tauopathy, and in-depth exploration of this signaling and regulation may provide valuable strategies for AD treatment.

In addition, tau also undergoes both *N*-linked glycosylation (*N*-glycosylation) [171] and *O*-linked *N*-acetylglucosaminylation (*O*-GlcNAcylation) [172], which have been proposed to affect tau phosphorylation and aggregation [173]. As discussed above, a reciprocal relationship has been found between O-GlcNAcylation and phosphorylation on tau [43]. Increases in tau O-GlcNAcylation inhibit tau aggregates and neuronal cell loss [174], indicating that O-GlcNAcylation modification can protect AD progression. O-GlcNAcylation levels are reduced in the cortex and hippocampus of AD individuals and in vivo and in vitro models of AD [175]. The reduced O-GlcNAcylation is related to mitochondrial dysfunction and neurodegeneration [175], which may be associated with neuroinflammation. Interestingly, the R47H variant of TREM2, which is expressed by microglia and linked to innate immunity, has been found to present an altered glycosylation pattern and decreased stability compared with wild-type TREM2 [176], indicating a potential link between microglial neuroinflammation and glycosylation [177, 178]. However, the direct correlation between neuroinflammation and glycosylation and how it regulates tau post-translational modification and tau pathology remains to be explored.

Abnormal acetylation of tau on lysine residues spans the microtubule-binding repeat region (MTBR), and this modification alone is sufficient to induce tau pathology and neurodegeneration [51]. Tau is acetylated by the lysine acetyltransferase p300 and its close homolog CREB-binding protein (CBP) [48]. The expression and activity of p300 are increased in the brain of AD patients [179], and the dysregulation of p300/CBP promotes tau acetylation, which could aggravate tau accumulation and pathology [46, 48]. Inhibiting p300 with salsalate, a non-steroidal anti-inflammatory drug, could induce tau deacetylation, preserve tau axonal localization, and protect mice from neurodegeneration [51]. The histone deacetylase 6 (HDAC6) has also been implicated in tau deacetylation/acetylation [180]. Cohen et al. reported that tau is a substrate of HDAC6, and the inhibition of HDAC6 increases tau acetylation [47]. HDAC6 also inhibits tau hyperphosphorylation within the MTBR [181]. However, other studies found that under neuroinflammatory stress, deletion or inhibition of HDAC6 suppresses mislocalization and neuritic aggregation of tau through a matrix metalloproteinase (MMP-9)-mediated mechanism [182]. HDAC6 inhibitors are being developed to treat immune and inflammatory diseases, including human immunodeficiency virus (HIV)/acquired immunodeficiency syndrome (AIDS) [183]. HDAC6 knockdown attenuates reactive oxygen species (ROS) generation, NADPH oxidase activation, and neuroinflammation response in HIV-1 transactivator of transcription (Tat)-stimulated astrocytes [184]. In addition, Nox2 knockdown suppresses HIV-1 Tat-induced HDAC6 expression and subsequent upregulation of pro-inflammatory chemokines [184], indicating a possible link between neuroinflammation and mediation of HDAC6. All these findings suggest that neuroinflammation-involved regulation of tau acetylation may be a potential therapeutic strategy to ameliorate tau pathology-involved neurodegeneration.

### Role of neuroinflammation in the propagation of tau

A large number of studies have explored the mechanisms of tau transmission between neurons and its impact on tau pathology. Here, we review the interplay between neuroinflammation and tau transmission. Emerging studies have indicated a possible interaction between the prion-like features of tau protein and the neuroinflammatory response in tau transmission and pathology [185, 186], although their causal relationship is currently uncertain. Microglia have been strongly implicated as a pivotal player and play a complex role in the propagation of tau pathology. In the brain of AD patients, microglial activation and tau accumulation propagate spatially in parallel, following brain circuits and staging of tau pathology [187]. Microglia can phagocytose and degrade pathologic tau, neuronal synapses, or whole live neurons, although not very efficiently [188, 189]. In addition, sustained reactive or senescent microglia become hypofunctional and release seed-competent tau, leading to the exacerbated spread of tau pathology [190].

Microglia sense pathological tau species via various surface receptors to trigger phagocytosis and/or degradation. Tau can bind to microglial CX3CR1 and initiate the internalization and degradation of tau [191]. CX3CL1 competes with tau for binding to CX3CR1, leading to a decrease in the internalization of tau. In addition, phosphorylated tau at Ser396 exhibits reduced binding affinity to CX3CR1 [191]. These findings indicate that CX3CL1/CX3CR1 axis plays a crucial role in tau phagocytosis and degradation by microglia. In addition, several studies have shown that tau seeds taken up by microglia can activate microglia, further exacerbating tau propagation and pathology [186, 192, 193]. Jin et al. reported that microglia uptake exogenous monomeric tau in parallel via two surface receptors, LRP1 and TREM2, and then induce nuclear factor NF-κB activation in microglia through two different pathways, LRP1/polyglutamine binding protein 1 (PQBP1)/cyclic GMP–AMP synthase (cGAS)/Stimulator of interferon genes (STING) and TREM2/extracellular signal-regulated kinase (ERK)/Phosphoinositide 3-kinase (PI3K) pathways, respectively (Fig. 1), ultimately triggering neuronal death [192]. Microglial NF-κB pathway activated by tau can exacerbate the processing and release of pathological tau with seeding activity. In contrast, deficiency or inhibition of NF-κB reduces the seeding and spread of tau inclusions in tauopathy mice [193]. However, another study revealed that TREM2 knockout or its R47H variant decreases microgliosis around Aβ plaques and enhances the propagation of tau aggregates [70]. This may be related to the different types of microglia [194]. In addition, the microglial NLRP3 inflammasome is also involved in the pathological propagation of tau. The aggregated tau seeds activate NLRP3/apoptosis-associated speck-like protein containing a CARD (ASC) inflammasome in microglia, following microglial uptake and lysosomal sorting of tau seeds [186]. Administration of NLRP3 inhibitor or ASC deficiency reduces the exogenously or non-exogenously seeded tau pathology, highlighting the promotion of NLRP3/ASC axis activation on the propagation of tau seeds [186]. In addition, exosomes are involved in the microglia-mediated pathological propagation of tau. Asai et al. demonstrated that depleting microglia or inhibiting microglial exosome synthesis reduces tau propagation from the entorhinal cortex to the dentate gyrus in the adeno-associated virus (AAV)/tau-injection P301S tauopathy mice [80] (Fig. 1). Suppression of P2X purinoceptor 7 (P2RX7)-induced exosome secretion from microglia attenuates misfolded tau aggregates in the hippocampus and improves cognitive deficits of P301S mice [195]. Furthermore, Zhu et al. reveal that TREM2 deficiency exacerbates pathological tau propagation via microglial exosomes [196]. These findings suggest that elucidating the mechanisms by which microglia participate in tau propagation may provide new strategies for the intervention of tau pathology.

In addition to microglia, astrocytes contribute to the spreading of tau pathology. It has been shown that astrocytic tau pathology occurs in AD and other tauopathies [197]. The aberrant tau in astrocytes may also come from the phagocytosis of neuronal debris and dystrophic synapses containing tau aggregates [198, 199]. TFEB induces astrocytic trafficking of tau fibrils via macropinocytosis, possibly through interaction with heparan sulfate proteoglycans (HSPGs) [200, 201]. Overexpression of transcription factor EB (TFEB, a regulator of lysosomal biogenesis) in astrocytes promotes tau fibril species uptake and lysosomal activity as well as attenuates tau spreading and pathology in the hippocampus of P301S tauopathy mice [201] (Fig. 1). Astrocytes can internalize tau monomers or fibrils through mechanisms independent of HSPGs [202]. The integrin αV/β1 receptor interacts with tau monomers or fibrils and mediates tau uptake in primary astrocytes. The binding of tau fibrils to astrocyte αV/β1 activates integrin signaling, resulting in NF-κB activation, leading to an increase of pro-inflammatory cytokines and chemokines, and induction of expression of neurotoxic astrocytic markers [203]. These findings suggest that phagocytized tau by astrocytes can be degraded and/or induce astrocytic activation. Whether astrocytic tau is secreted to the outside of cells as exosome remains controversial [80, 204]. As discussed above, we indicated that CMKLR1 expressed on neurons affects tau phosphorylation via mediating tau seeding [14] (Fig. 1), and how CMKLR1 on glial cells contributes to tau propagation needs investigation. In addition, it has been found that astrocyte-derived exosomes drive neurodegeneration of AD through the acceleration of Aβ aggregation in vivo [205]. Whether astrocytes can promote tau pathology via secreting exosomes still requires clinical evidence and experiment verification.

Thus, these results suggest that the interaction of pathological tau with glial cells promotes the propagation of pathological tau and AD progression. Modulating the activity of glial cells, the expression of surface receptors, the activation of inflammation-related signaling pathways, or the release of exosomes to interfere with their promotion of tau spread may provide potential strategies for AD treatment.

## Advances in AD drug development focused on tau pathology and neuroinflammation

Current pharmacologic treatments for AD are three cholinesterase inhibitors (donepezil, rivastigmine, and galantamine), one N-methyl-D-aspartate (NMDA) receptor antagonist (memantine), and two DMT anti-Aβ antibody drugs (aducanumab and lecanemab). These cholinesterase inhibitors and NMDA receptor antagonist memantine only temporarily relieve the symptoms of AD patients but do not delay the progression of the disease [206, 207]. For the past two decades, the therapeutic approaches have focused on developing DMT drugs, particularly those targeting Aβ. However, with the failures or unsatisfactory efficacy of drugs anti-Aβ [9, 208–211], the development of drugs targeting other targets is increasing, such as anti-tau pathology and anti-neuroinflammation [212]. According to the report by Cummings et al., among the 143 drugs in trials in the AD drug development pipeline as of January 2022, DMT drugs account for 83.2% [212]. There are 20 (16.8%) agents targeting Aβ, 13 (10.9%) targeting tau, 28 (19.3%) targeting inflammation, and 19 (16%) targeting synaptic plasticity or neuroprotection.

### Tau pathology as a therapeutic target

There are no drugs yet approved to treat tauopathies. Currently, tau-targeted disease-modifying therapies developed for AD or other tauopathies mainly include mediators of tau post-translational modifications, anti-tau immunotherapy (active and passive), tau aggregation inhibitors, microtubule stabilizers, and gene therapy. We will primarily review drugs in clinical trials targeting tau post-translational modifications and anti-tau immunotherapy (Table 1). Anti-tau immunotherapy aims to target tau phosphorylation, aggregation, and propagation, and clear extracellular and/or intracellular pathological tau.

**Table 1:** Representative AD drug candidates targeting tau and neuroinflammation under clinical study

Candidate	Target	Mechanism	Study phase	Current status (ClinicalTrials.gov Identifier)	Sponsor	Outcome
Tideglusib	Tau	GSK-3β inhibitor	Phase 2	Completed; (NCT00948259)	Noscira SA	No cognitive or functional benefits
			Phase 2	Completed; (NCT01350362)		
Lithium	Tau	GSK-3β inhibitor	Phase 2	Completed; (NCT00703677)	Westat	Stopped due to poor tolerability
			Phase 2	Recruiting; (NCT05423522)	Medesis Pharma SA	Ongoing, ECD: Jun 2024
LY3372689	Tau	O-GlycNAcase inhibitor	Phase 2	Active, not recruiting; (NCT05063539)	Eli Lilly and Company	Ongoing, ECD: Jun 2024
Salsalate	Tau	Tau acetylation inhibitor	Phase 1b	Unknown; (NCT03277573)	Adam Boxer	Unknown
AADvac1	Tau	Anti-tau active vaccine	Phase 2	Completed; (NCT02579252)	Axon Neuroscience SE	No cognitive benefits
ACI-35.030	Tau	Anti-tau active vaccine	Phase 1/2	Active, not recruiting; (NCT04445831)	AC Immune, Janssen	Ongoing, ECD: Oct 2023
Gosuranemab (BIIB092)	Tau	Anti-tau monoclonal antibody	Phase 2	Terminated; (NCT03352557)	Biogen	Lack of efficacy
Tilavonemab	Tau	Anti-tau monoclonal antibody	Phase 2	Completed; (NCT02880956)	AbbVie	No cognitive or functional benefits
Zagotenemab	Tau	Anti-tau monoclonal antibody	Phase 2	Completed; (NCT03518073)	Eli Lilly and Company	Miss its primary endpoint
Semorinemab (RO7105705)	Tau	Anti-tau monoclonal antibody	Phase 2	Active, not recruiting; (NCT03828747)	Genentech	Ongoing, ECD: Aug 2023
			Phase 2	Terminated; (NCT03289143)		No clinical efficacy
BIIB076	Tau	Anti-tau monoclonal antibody	Phase 1a	Completed; (NCT03056729)	Biogen	Unknown
E2814	Tau	Anti-tau monoclonal antibody	Phase 2	Active, not recruiting; (NCT04971733)	Eisai	Ongoing, ECD: Sep 2024
			Phase 2/3	Recruiting; (NCT05269394)	Washington University School of Medicine	Ongoing, ECD: Oct 2027
Lu AF87908	Tau	Anti-tau monoclonal antibody	Phase 1a	Recruiting; (NCT04149860)	Lundbeck	Ongoing, ECD: Jun 2023
JNJ-63733657	Tau	Anti-tau monoclonal antibody	Phase 1a	Completed; (NCT03689153)	Janssen Research and Development, LLC	Well-tolerated in Phase I
			Phase 2	Recruiting; (NCT04619420)		Ongoing, ECD: Nov 2025
PNT001	Tau	Anti-tau monoclonal antibody	Phase 1a	Completed; (NCT04096287)	Pinteon Therapeutics, Inc	Well-tolerated in Phase I
APNmAb005	Tau	Anti-tau monoclonal antibody	Phase 1	Recruiting; (NCT05344989)	APRINOIA Therapeutics	ECD: Jan 2023, no results reported
Bepranemab (UCB0107)	Tau	Anti-tau monoclonal antibody	Phase 1a	Completed; (NCT03605082)	UCB Biopharma	No drug-related adverse events were reported
			Phase 2	Active, not recruiting; (NCT04867616)		Ongoing, ECD: Jul 2025
Neflamapimod (VX-745)	Inflammation	p38 MAPK-α inhibitor	Phase 2	Completed; (NCT03402659)	EIP Pharma	Miss its primary endpoint of improving episodic memory
			Phase 2	Unknown; (NCT03435861)	University Hospital, Fondation Plan Alzheimer	Unknown
			Phase 2	Completed; (NCT04001517)	EIP Pharma	Improve cognitive and motor function
MW150	Inflammation	p38 MAPK-α inhibitor	Phase 2	Not yet recruiting; (NCT05194163)	Neurokine Therapeutics, Columbia University, NIA	Ongoing, ECD: Nov 2024
MW151	Inflammation	p38 MAPK-α inhibitor	Phase 1	Completed; (NCT04120233)	Duke Clinical Research Institute, NIA	No drug-related adverse events were reported
Nilotinib	Tau, Inflammation	Tyrosine kinase inhibitor; promotes clearance of Aβ and tau	Phase 2	Completed; (NCT02947893)	Georgetown University	Safe and achieve pharmacologically relevant CSF concentrations
			Phase 3	Not yet recruiting; (NCT05143528)	KeifeRx	Ongoing, ECD: Jun 2026
NE3107 (HE3286)	Inflammation	Binds to ERK1/2, inhibit ERK/NF-κB pathway	Phase 3	Active, not recruiting; (NCT04669028)	BioVie Inc	Ongoing, ECD: Oct 2023
AZP2006	Tau, Inflammation	The neurotrophic factor progranulin enhancer	Phase 2a	Completed; (NCT04008355)	AlzProtect SAS	No results reported
Dasatinib + Quercetin	Tau, Inflammation	Tyrosine kinase inhibitor (dasatinib); flavonoid (quercetin); reduce senescent cells and tau aggregation	Phase 1/2	Completed; (NCT04063124)	The University of Texas Health Science Center at San Antonio, Mayo Clinic	Safe and tolerable in this trial
			Phase 1/2	Enrolling by invitation; (NCT04785300)	Mayo Clinic	Ongoing, ECD: Dec 2023
GV-971 (Sodium oligomannate)	Amyloid-Related, Inflammation	Remodel the gut microbiota, reduce microglial activation and immune responses	Phase 3	Suspended; (NCT04520412)	Green Valley (Shanghai) Pharmaceuticals	The trial was affected by the COVID-19

The candidate information was collected from the clinical trial database (clinicaltrials.gov) provided by the United State National Library of Medicine and the Alzforum (alzforum.org) (accessed on March, 2023). *AD* Alzheimer’s Disease, *COVID-19* coronavirus disease 2019, *CSF* cerebrospinal fluid, *ECD* estimated completion date, *ERK* extracellular signal-regulated kinase, *GSK-3β* glycogen synthase kinase-3β, *MAPK* mitogen-activated protein kinase, *NIA* National Institute on Aging, *NF-κB* nuclear factor kappa B

### Mediators of tau post-translational modifications

Various kinases are known to induce tau hyperphosphorylation, such as GSK-3β, a major tau kinase. These kinases are hyperactivated during the progression of AD and other tauopathies [19], indicating that the development of their mediators may be an effective treatment for these diseases. Two GSK-3β inhibitors, lithium and tideglusib, have been tested [213]. Forlenza et al. reported that long-term lithium treatment at subtherapeutic doses attenuates cognitive and functional decline in amnestic MCI (ClinicalTrials.gov Identifier: NCT01055392) [214]. However, this dose of lithium treatment still has safety issues, such as side effects and tolerability (NCT01055392, NCT00703677) [215]. Another clinical trial evaluating the safety and efficacy of a lower dose of lithium in patients with mild to severe AD just started in June 2022 and is estimated to be completed in June 2024 (NCT05423522). Tideglusib, a thiadiazolidinone derivative, acts as a non-ATP competitive GSK-3β inhibitor. Tideglusib has been reported to reduce tau phosphorylation and has anti-inflammatory effects in animal models [216, 217]. The clinical studies in patients with mild-to-moderate AD (NCT01350362, NCT00948259) or PSP (NCT01049399) patients treated with tideglusib were negative on the clinical outcomes [218]; however, a subgroup analysis of PSP patients showed reduced brain atrophy [219].

There is a reciprocal relationship between O-GlcNAcylation and tau phosphorylation [43]. Inhibitors of the O-GlcNAcase enzyme (OGA)-like thiamet G increase tau glycosylation, reduce NFTs and neuronal cell death, improve motor behavior, and prolong survival of tau transgenic mice [174, 220, 221]. Three OGA inhibitors, ASN90, ASN51, and LY3372689, are currently in clinical trials. They have shown well safety and tolerance in phase 1 clinical trials [222, 223]. Phase 2/3 clinical trials of these inhibitors for the treatment of AD and PSP are ongoing (NCT05063539, [223]). Salsalate, which we discussed above, induces tau deacetylation. A clinical trial has shown that it does not affect disease progression in PSP patients (NCT02422485). A phase 1 clinical trial testing the safety, tolerability, and cognitive ability of salsalate in patients with mild to moderate AD (NCT03277573) has not yet published the results.

### Anti-tau immunotherapy

In addition to the small molecule drugs targeting tau post-translational modifications discussed above, active and passive immunotherapies have been developed to neutralize and clear toxic tau species to reduce tau phosphorylation, aggregation, and dissemination. Active immunization, such as vaccination, can cause the body to produce an antibody-like response. AADvac1 and ACI-35/ACI-35.030 are anti-tau active vaccines for AD and other tauopathies currently in clinical trials [224, 225]. AADvac1 is a synthetic peptide corresponding to amino acids 294 to 305 of the tau sequence, and ACI-35 consists of 16 copies of a synthetic tau fragment phosphorylated at Ser396 and Ser404 sites [225]. Phase 1 clinical data show that AADvac1 has well safety, tolerability, and immunogenicity in patients with mild to moderate AD (NCT02031198, NCT01850238) [226]. However, the treatment of this vaccine did not slow cognitive and functional decline in mild AD patients in the phase 2 clinical trial (ADAMANT, NCT02579252), although it shows the high immunogenicity [227]. A phase 1 pilot trial investigating the effect of AADvac1 in patients with non-fluent primary progressive aphasia (AIDA) has yet to publish results (NCT03174886). ACI-35 treatment was reported to produce a weak immune response in a phase 1 study [228]. Therefore, a redesigned version, ACI-35.030, was indicated to elicit a stronger immune response [228]. A phase 1/2 clinical trial to test its safety and immunogenicity in patients with early AD is undergoing and expected to be completed in 2023 (NCT04445831).

Another immunotherapeutic approach is administering preformed anti-tau antibodies direct against different tau epitopes, also known as passive immunotherapy. Passive immune antibody drugs have the advantages of low risk of adverse effects to immunogenicity and more specificity for targeted epitopes. A total of 12 tau antibodies have entered clinical trials, but half of tau antibody trials have been terminated due to poor clinical efficacy for AD or PSP. These antibodies include gosuranemab [229], tilavonemab [230], zagotenemab [231], semorinemab [232], RG7345 [233], and BIIB076 [234]. These antibodies mainly bind the N- or C-terminal epitopes of tau. Recent clinical evidence indicates that antibodies targeting the microtubule-binding region (MTBR, residues 224 to 369) or phosphorylated epitopes around the center of tau are more likely to prevent the propagation of pathogenic aggregated tau [235, 236]. Antibodies binding MTBR such as E2814 [237] and antibodies targeting phosphorylated epitopes of MTBR or mid-domain of tau, including JNJ-63733657 [238], Lu AF87908 [239], PNT001 [225], bepranemab (UCB0107) [240], and APNmAb005 [241] have been shown to prevent tau pathology and dissemination in preclinical animal experiments. The antibody Lu AF87908 also regulates the uptake and lysosomal function to clear pathological tau by interacting with the IgG antibody receptor FcγR in primary microglia cultures [242], indicating that anti-tau antibodies may induce microglial clearance of tau and reduce tau propagation. Furthermore, the antibodies bepranemab, semorinemab, JNJ-63733657, E2814, and PNT001 showed good safety and tolerability in the phase 1 clinical trial (NCT03605082, NCT02820896, NCT03689153, NCT04231513, and NCT04096287). The phase 2/3 clinical trials to test the efficacy of E2814, bepranemab, semorinemab, and JNJ-63733657 in patients with AD or other tauopathies are ongoing (NCT04971733, NCT04867616, NCT03828747, and NCT04619420). Since E2814 is designed to target pathologic tau and can attenuate aggregated tau spreading in preclinical experiments [237], and lecanemab, an anti-Aβ antibody approved through the FDA accelerated approved process for AD treatment, can reduce the brain amyloid and slow the rate of cognitive decline in patients with early AD [10]. A phase 2/3 clinical study is underway to evaluate the cognition and clinical efficacy of E2814 alone, lecanemab alone, and the combination of these two drugs in participants with autosomal dominant AD (DIAN-TU, NCT05269394).

### Therapeutically targeting tau through modulating neuroinflammation

A total of more than 50 inflammation-related agents have entered clinical trials for AD treatment, and 22 are currently undergoing mainly targeting the inflammatory (especially neuroinflammatory) response process that may result in neurodegeneration, such as regulating the function of microglia and the immune system, the activity of inflammatory response-related kinases/pathway, and the expression and release of pro-inflammatory factors. Here, we will mainly review neuroinflammation-related drugs in clinical trials that may also have modulatory effects on tau pathology (Table 1).

As mentioned above, p38α MAPK is a key kinase involved in microglia-induced neuroinflammation leading to tau pathology [135, 137], and this kinase can also directly regulate tau phosphorylation [39]. Recently, the p38α inhibitors such as neflamapimod have attracted attention as potential treatments for neurodegenerative diseases, including AD. A multi-center phase 2 clinical trial showed that although a 24-week treatment with neflamapimod did not ameliorate episodic memory in individuals with mild AD, neflamapimod treatment significantly reduces CSF total tau and tau phosphorylated at threonine 181 (pTau181) compared to the placebo group (NCT03402659). The results of this trial suggest that a longer study of neflamapimod at higher dose levels is needed to assess the effect on AD progression [243]. Notably, a phase 2a clinical study suggested that neflamapimod treatment improved cognitive and motor functions in patients with mild-to-moderate dementia with Lewy bodies (DLB) [244] (NCT04001517). These results indicate that inhibiting p38α MAPK to improve tau pathology may be one of the most promising strategies for AD treatment. Other p38α inhibitors, such as MW150 [245], MW151 [246], and MW189 (the intravenous formulation of MW151), all showed good safety and tolerability in phase 1 clinical trials (NCT04120233, NCT02942771) [247]. A phase 2 study of MW150 in patients with mild to moderate AD is underway (NCT05194163).

Nilotinib is a small molecule tyrosine kinase inhibitor with potent p38α inhibitory activity [248]. It modulates the immune profiles of glial cells and induces phosphorylated tau clearance, although not more efficiently than Aβ [249, 250]. A phase 2 clinical trial in 37 patients with mild to moderate AD (NCT02947893) showed that nilotinib was well-tolerated and reduced CSF pTau181 at 6 months compared to the placebo group [251].

NE3107, which reached phase 3 (NCT04669028), is a derivative of β-androstenediol. It binds to ERK and inhibits the activation of ERK/NF-κB signaling. NE3107 orally enters the brain. It has anti-neuroinflammatory and insulin-sensitizing properties, which make it attractive in AD treatment [252, 253]. A phase 2 clinical study presented at the 2022 clinical trials on Alzheimer’s disease (CTAD) conference showed that NE3107 was associated with improvement in CSF p-tau in patients with MCI or mild dementia [254]. A phase 3 trial to evaluate the safety and efficacy of this agent in subjects with mild to moderate probable AD (NCT04669028) started in August 2021 and is estimated to be completed in October 2023.

In addition, AZP2006, a small molecule to prevent the growth factor progranulin (PGRN) cleavage and promote its secretion, has also been reported to inhibit tau phosphorylation and neuroinflammation in a preclinical study [255]. The chronic treatment of AZP2006 attenuates the cognitive impairments and neuronal synaptic damage, accompanied by significant decreases in microglial activation, proinflammatory cytokine release, and tau hyperphosphorylation in the brains of AD and aging model mice. Further mechanistic studies demonstrated that AZP2006 binds to PSAP (a cofactor of PGRN) and inhibits the TLR9-driven signaling to reduce pro-inflammatory responses. These results indicate the potential of AZP2006 as a novel strategy for the treatment of AD and other tauopathies [255]. One phase 2 trial is ongoing to evaluate the safety, tolerability, pharmacokinetics, and effect of AZP2006 in patients with PSP (NCT04008355).

Another promising new therapy targeting tau pathology and neuroinflammation is the combination treatment of dasatinib and quercetin. Dasatinib is a cancer drug that inhibits Src tyrosine kinase. Quercetin inhibits the anti-apoptotic protein Bcl-xL and has anti-inflammatory activity. This drug combination eliminates the senescent cells (also known as “senolytic therapy”), reduces tau pathology, amyloid load, and neuroinflammation, and ameliorates cognitive impairment in preclinical studies [256, 257]. Dasatinib orally enters the brain, and the drug combination appears safe and tolerable in phase 1/2 clinical studies [258, 259]. Several clinical studies are underway to test this drug combination's efficacy in treating AD.

Sodium oligomannate (GV-971), a derivative of marine algae oligosaccharides, received conditional approval in China to treat mild to moderate AD in 2019 [260]. The preclinical data showed that GV-971 remodels the gut microbiota and the associated accumulation of phenylalanine and isoleucine, reduces microglial activation, immune responses, Aβ plaque deposition, and tau phosphorylation in the brain, and improves the cognitive impairment in 5xFAD mice [261]. GV-971 has also been demonstrated to reverse cognitive deficits in patients with mild to moderate AD in the phase 2/3 clinical trial in China (NCT02293915) [262]. Although the long-term efficacy and safety of GV-971 need to be further verified, these results highlight that multi-target drugs against multiple AD pathological changes may be a more potential therapeutic strategy.

## Conclusions and perspectives

Over the past few decades, accumulated efforts in basic and clinical research in the AD field have improved our understanding of the multifactorial nature of AD pathogenesis. It is particularly encouraging that two DMT drugs targeting Aβ have recently received accelerated FDA approval for AD treatment. However, the efficacy of these drugs is controversial and needs further validation. The most likely reason for the failure of drugs targeting Aβ may be that Aβ pathology in AD does not always correlate with cognitive decline.

Tau has received increasing attention as a potential alternative therapeutic target, since evidence indicates that tau pathology is more associated with cognitive degradation. To date, there are no tau-focused drugs approved by FDA. Still, several agents targeting tau post-translational modification and dissemination have recently entered clinical trials for treating AD and other tauopathies. Accumulating evidence indicates that neuroinflammation may be the third pathological feature of AD and plays an integral role in AD pathogenesis and the promotion of cognitive impairment. In recent years, growing findings of fundamental research have demonstrated the complex interplay between neuroinflammation and tau pathology, as summarized above. These findings suggest that in the early stage of AD, a moderate inflammatory response may alleviate tau pathology, such as activated microglia promoting the clearance of tau seeds. While in the middle and late stages of AD, sustained and increased inflammatory responses in glial cells and neurons are pivotal cellular drivers and regulators of the exacerbation of tau pathology, further contributing to its worsening by promoting inflammatory responses. This vicious circle aggravates the pathological progression of AD. There are temporal and spatial dynamic regulatory processes between tau pathology and inflammation as the disease progresses. Further elucidation of these dynamic regulatory mechanisms will provide essential insights into AD pathogenesis and drug development.

Furthermore, current studies indicate that targeting only a single therapeutic target may not be able to reverse the pathological process of AD. As mentioned above, multi-target therapies (including multi-target single drugs such as the p38α inhibitors and single or multiple target multi-drug combination such as the combination of dasatinib and quercetin) targeting tau pathology and neuroinflammation, or simultaneously targeting Aβ pathology, which is currently undergoing clinical trials, may offer hope of reversing the course of AD and even curing it. These therapeutic strategies have shown good safety and tolerability, and clinical trials of their therapeutic effects are underway. In summary, both basic research and clinical trials suggest that targeting multiple pathologies and precise treatment strategies will be the trend of future drug development for AD and other tauopathies. They will be more likely to bring breakthroughs in the treatment of these diseases.

## Data Availability

All relevant data are included in this review.

## Abbreviations

AD: Alzheimer’s disease
Aβ: β-Amyloid
ApoE4: Apolipoprotein E4
cGAS: Cyclic GMP–AMP synthase
CMKLR1: Chemerin chemokine-like receptor 1
CX3CL1: Chemokine (C–X3–C motif) ligand 1
CX3CR1: CX3C motif chemokine receptor 1
FPR2: Formyl peptide receptor 2
GSK-3β: Glycogen synthase kinase-3 beta
IL-1β: Interleukin-1β
HSPGs: Heparan sulfate proteoglycans
LRP1: Low-density lipoprotein receptor-related protein 1
MAPK: Mitogen-activated protein kinase
NF-κB: Nuclear factor kappa B
NFTs: Neurofibrillary tangles
NLRP3: NLR family pyrin domain-containing protein 3
P2RX7: P2X purinoceptor 7
PHFs: Paired helical filaments
STING: Stimulator of interferon genes
TNF-α: Tumor necrosis factor α
TREM2: Triggering receptor expressed on myeloid cells 2
